# Validation and Cultural Adaptation of the Serbian Version of the Pediatric Outcome Data Collection Instrument (PODCI) in Children with Obstetrical Brachial Plexus Lesion

**DOI:** 10.3390/medicina58060807

**Published:** 2022-06-15

**Authors:** Jasna Stojkovic, Dragana Cirovic, Ivana Petronic, Dejana Stanisavljevic, Sinisa Ducic, Branislav Jovanovic, Jelena Pejanovic Jovanovic, Tamara Filipovic, Slobodan Subotic, Dejan Nikolic

**Affiliations:** 1 Faculty of Medicine, University of Belgrade, 11000 Belgrade, Serbia; stojkovic.jasna@yahoo.com (J.S.); cirovicdragana@yahoo.com (D.C.); ivana.pm@live.com (I.P.); sdejana8@yahoo.com (D.S.); sinisaducic@gmail.com (S.D.); branislav.jovanovic@udk.bg.ac.rs (B.J.); jelena.pejanovic@udk.bg.ac.rs (J.P.J.); tamarabackovic@gmail.com (T.F.); 2 Department of Physical Medicine and Rehabilitation, University Children’s Hospital, Tirsova 10, 11000 Belgrade, Serbia; 3 Department of Pediatric Surgery, University Children’s Hospital, 11000 Belgrade, Serbia; 4 Institute for Rehabilitation, 11000 Belgrade, Serbia; 5 College of Health Studies “Milutin Milankovic”, 11000 Belgrade, Serbia; slobasubotic@yahoo.com

**Keywords:** validation, temporal stability, questionnaire, brachial plexus lesion, children

## Abstract

*Background and objectives:* Pediatric Outcome Data Collection Instrument (PODCI) is among the most described scales developed to evaluate the physical status of children and adolescents with various musculoskeletal disorders. We aimed to translate PODCI from English to Serbian, culturally adopt items and domains, evaluate the temporal stability, internal consistency and the test–retest reliability of PODCI_SR_ in children with obstetrical brachial plexus lesion (OBPL), and finally, to test the construct validity of PODCI_SR_ against muscular manual test (MMT) *Materials and Methods:* The study included 48 eligible participants aged between 2 and 10 years with OBPL. The MMT was used to test the construct validity. *Results:* There were no significant differences (*p* > 0.05) between test and retest for all PODCI_SR_ domains. Correlations for all tested domains with MMT were statistically significant except for biceps muscle and domains II and IV. Cronbach’s alpha value of the Global Functioning Scale was good and equaled 0.838 for test and 0.832 for retest session. Cronbach’s α was more than 0.600 for all PODCI_SR_ domains except for Domain II and for Domain IV. The observed Test–Retest ICC for all PODCI_SR_ domains scores ranged from 0.899 to 0.996. *Conclusion:* The Serbian version of PODCI (PODCI_SR_) was successfully translated and transculturally adopted. It has satisfactory temporal stability, construct validity and test–retest reliability as well as relevant internal consistency.

## 1. Introduction

Obstetrical brachial plexus lesions (OBPL) are still a burden in neonatal and pediatric practice, and they present with a frequency of 1–4 per 1000 newborns [1]. Aside advances in obstetrics, previous reports stated that the incidence did not decline during the last decades [2]. Despite the fact that the majority of children with OBPL have satisfactory recovery, there are around 20–30% of them that are permanently functionally challenged in various ways [1].

In the past decade, many activity-based physical-function scales have been developed to evaluate physical status of children and adolescents with musculoskeletal disorders [3,4]. One of the most described scales is “The Pediatric Outcome Data Collection Instrument” (PODCI). It was developed by the American Academy of Orthopedic Surgeons (AAOS), Pediatric Orthopedic Society of North America (POSNA), the American Academy of Pediatrics (AAP) and Shriner’s Hospitals. It is used to evaluate the functional limitation, and to assess therapeutic needs and changes after the treatment in 2 to 18 years old children and adolescents who have orthopedic problems [5]. PODCI has three forms: parent report for children 2–10 years and parent and self-reports for adolescents 11–18 years. It is used for different musculoskeletal disorders: Cerebral Palsy (CP), Legg-Calves-Perthes, Scoliosis, Juvenile Rheumatoid Arthritis, Osteogenesis imperfecta, etc. So far, PODCI has been translated into multiple languages (Dutch, Turkish, Brazilian Portuguese) [6,7,8], but it is not translated into Serbian. The PODCI questionnaire was previously used for the evaluation of upper extremity functioning along with global functioning in children with OBPL [9]. Furthermore, the PODCI was shown to be effective for the evaluation of the individuals’ perceived quality of life after the treatment [9].

The first aim of this study was to translate PODCI from English to Serbian and to culturally adopt the items and domains bearing in mind the possible cultural differences. The second aim was to evaluate the temporal stability, internal consistency and the test–retest reliability of PODCI_SR_ in children with OBPL. Finally, the third aim was to test the construct validity of PODCI_SR_ against muscular manual test (MMT).

## 2. Material and Methods

The study included 48 eligible participants aged between 2 and 10 years, who were referred to University Children’s Hospital for the diagnosis and treatment of obstetrical brachial plexus lesion. At admission, children were evaluated by a board-certified specialist of Physical Medicine and Rehabilitation and a board-certified specialist of Pediatric Surgery. The diagnostic protocol was introduced and included: clinical and neurological examination, radiographic imaging of both arms and shoulders to exclude possible fractures, joint dislocations and bone tissue infections. Furthermore, participants’ blood was drawn from a vein to check blood cells count, C reactive protein (CRP) and erythrocyte sedimentation (SE). The final diagnostic tool that was performed included electromyoneurographic (EMNG) evaluation for the detection of nerve lesions, the level and the degree of the lesion.

Patients with secondary fractures, secondary nerve lesions, vascular dysfunctions, and bilateral brachial plexus injuries were excluded from the study.

Prior to inclusion in the study, parents or legal guardians were informed about the study protocol and consent was obtained. The study was approved by the institution ethics committee and followed the principles of good clinical practice and the Helsinki declaration.

### 2.1. Clinical and Neurological Examination

The affected hands of eligible participants were evaluated. Presence of active movements in all joints on the affected hand were screened and the strength was measured by the Muscular Manuel Test (MMT). The MMT was categorized into 6 categories: 0, no muscle contractility; 1–presence of slight contractility but without obvious movement: 2, the presence of movement but without the gravity force: 3, the presence of movement against gravity but without resistance force: 4, the presence of movement against gravity with partial resistance force: 5, the presence of movement against gravity with full resistance force [10]. The passive range of motion of the affected hand was further analyzed for every joint.

### 2.2. PODCI Questionnaire

Pediatric Outcome Data Collecting Instrument (PODCI) has 5 domains and one domain that represents total score of all domains, excluding happiness [8]. We used the parent-reported 2–10 years version (86 questions). The domains were as follows: Domain I, Upper extremity and physical function (8 items); Domain II, Transfer and basic mobility function (11 items); Domain III, Sports and physical function (21 items); Domain IV, Pain and comfort function (3 items); Domain V, Happiness function (5 items); Domain VI, Global function [8,11]. For this study we used PODCI_SR_ standardized scores of tested items [4].

### 2.3. PODCI Questionnaire Translation and Adaptation

For the adequate translation and cultural adaptation of the patients’ reported outcome measures [11] we used a forward–backward method [12,13] for the PODCI questionnaire. In Serbia, there is a lack of availability of professional translators with experience in translating parents’ reported outcomes measurements. Improper translation of the instruments undergoing validation process in different languages could be the cause of changes in the sensitivity and specificity of original instruments, potentially resulting in an inadequate comparability of responses between different populations [14]. Therefore, due to the aforementioned lack of professional translators or native English speakers in Serbia with experience in this specific topic, two lead authors first translated all the items separately. Each initial forward translation was distributed to the study investigators that are professional experts in the field of pediatrics and rehabilitation and are native Serbian speakers. Afterwards, the two preliminary translations were discussed, compared and merged to create a final version. For the backward translation to English, this version was sent to the bilingual professional individuals with spoken Serbian native language skills who had been living in an English-speaking country for more than 5 years. These professional individuals were not involved in the study. The differences between the forward and backward translations were compared and discussed and final pilot version of PODCI_SR-VER1_ was adopted at a meeting of the authors, and was then presented to five parents for feedback. The outcome was good, without complaints and no non-understandable questions. After the final meeting and achieved consensus of four board-certified specialists in Physical and Rehabilitation Medicine, the final version was adopted as PODCI_SR_. The final PODCI_SR_ questionnaire was presented to the tested subjects on two occasions (sessions) (test–retest) with a period of five days between the two testing sessions.

### 2.4. Statistical Analysis

The raw scores of PODCI_SR_ on both sessions (test and retest) were presented as mean values (MV), standard deviation (SD) and 95% Confidence Interval (CI). The statistical significance between the obtained values was evaluated by the Student paired *t*-test. For construct validity, the scores of each domain of PODCI_SR_ were correlated with MMT (separately for muscles: deltoid, biceps and triceps) using Spearman’s rho correlation coefficient (ρ). For internal consistency, Cronbach’s α was calculated for each domain of PODCI_SR_ on both sessions (test and retest). The values of 0.9 and above were considered excellent, 0.8–0.9 good, 0.7–0.8 acceptable, 0.6–0.7 questionable, and 0.5–0.6 poor for internal consistency [15]. For test–retest reliability, we used the intraclass correlation coefficient (ICC) for every domain of PODCI_SR_. The ICC was interpreted as poor (<0.40), moderate (0.40–0.59), good (0.60–0.74), and excellent (≥0.75) [15]. To assess the strength of association between the tested domains of PODCI_SR_ on both sessions (test and retest) we used Pearson‘s correlation coefficients. The significance was set as *p* < 0.05. All analyses were conducted using IBM SPSS statistical software (SPSS for Windows, release 21.0, SPSS, Chicago, IL, USA).

## 3. Results

In Table 1, the mean values of six domains of the test and the retest for the Serbian version of the PODCI questionnaire are presented. There were no significant differences between the two tested occasions for all domains.

The results of construct validity are presented in Table 2. Correlations for all tested domains with MMT for deltoid muscle were statistically significant, where the lowest correlation was for Domain IV (ρ = 0.361) and the highest for Domain V (ρ = 0.650). For MMT for biceps muscle, domains I, III, V and VI significantly correlated where lowest correlation was for Domain IV (ρ = 0.151) and highest from Domain V (ρ = 0.565). For MMT for triceps muscle, all domains significantly correlated where the lowest correlation was for Domain II (ρ = 0.333) and the highest was for Domain VI (ρ = 0.582).

Cronbach’s alpha value of the Global Functioning Scale was good and equaled 0.838 for the test and 0.832 for the retest session. Cronbach’s α was more than 0.600 for all PODCI_SR_ domains in the test session, except for Domain II and for Domain IV (Table 3). In the retest session, all PODCI_SR_ domains except Domain II and Domain IV were above 0.600. They ranged from 0.107 (Domain IV) to 0.934 (Domain V) in the test session and from 0.102 (Domain IV) to 0.926 (Domain V) in the retest session.

When Item 1 was removed from PODCI, Domain IV increased its Cronbach’s α value to 1.00 in both the test and retest sessions (Table 4).

The observed test–retest ICC was more than 0.750 for all PODCI_SR_ domains scores (ranging from 0.899 for Domain II to 0.996 for Domain V) (Table 3).

The Pearson correlation coefficients among different domains of PODCI_SR_ scores ranged from 0.136 to 0.901 on the test session and from 0.142 to 0.899 on the retest session (Table 5). The lowest values were noticed for correlations of Domain I with Domain IV (r = 0.136), on the test sessions and between the same domains on the retest session (r = 0.142).

Results of the temporal stability due to age of the patients are presented in Table 6. Temporal stability does not depend on age of the patients because there are no significant differences in the mean values between test and retest.

## 4. Discussion

The results of our study demonstrate that the correlations for all tested domains of PODCI_SR_ with MMT for deltoid muscle were statistically significant. Furthermore, Cronbach’s α was more than 0.600 for all PODCI_SR_ domains on test session, except for Domain II and for Domain IV and the observed test–retest ICC was more than 0.750 for all PODCI_SR_ domains. Additionally, we have shown that for PODCI_SR_, temporal stability does not depend on the age of the patients since there are no significant differences in the mean values between test and retest.

For the adequate process of transcultural adaptation, we aimed to follow idiomatic and semantic equivalence [16] between the original and translated PODCI versions. It should be stressed that transcultural adaptations are complex and sensitive, especially for the purpose of the validation of standardized questionnaires, thus scientists and collaborators should aim to preserve the sensitivity of items and domains [17]. For proper adaptation of the testing instrument, several aspects regarding translation should be taken into consideration, including cultural, idiomatic, linguistic and contextual aspects [18]. Improper translation of the instruments undergoing the validation process in different languages could be the cause of changes in the sensitivity and specificity of original instruments, potentially resulting in an inadequate comparability of responses between different populations [14].

In our study, after the final approval by the board of professionals for the translated and culturally adopted PODCI_SR_, despite minor cultural adaptations, no misinterpretation of tested items and generated domains during the feedback process were received. Therefore, we have assumed that the PODCI_SR_ version was properly translated with successful cultural adaptation of the tested items and domains. 

In our study, we have shown that there were lower scores for the domain of upper extremity and physical functioning of PODCI_SR_ compared to other domains that is in line with previous reports [9]. However, it should be taken into consideration that PODCI might be not that sensitive for the evaluation of shoulder function, particularly external rotation [19]. It is worth mentioning that the domain of Global functioning in this study was somewhat higher compared with the domains of Happiness and Pain and discomfort. The possible explanation for such a trend could be in the fact that our study population was aged between 2–10 years.

Considering the temporal stability of PODCI_SR_ in our study, we have demonstrated a satisfactory level for all domains since there were no significant differences in the mean values between both test sessions (test–retest). Obtaining the satisfactory temporal stability is important because numerous factors that might affect such parameter are taken into consideration including but not limited to: forgetting, dissonance reduction, reinterpretation, reduction of distortions due to initial emotional reactions, etc. [20]. Additionally, in our validation of PODCI_SR_, the Cronbach’s alpha values for the Global Functioning Scale domain and Upper extremity and physical function domain were good. This is of importance particularly in the evaluation of patients with OBPL, since these two domains could be relevant to a certain degree in the evaluation of one’s perceived quality of life [9].

To evaluate and test the construct validity of PODCI_SR_, we made comparisons with MMT, which was tested on three muscles separately. This measure describes how meaningful the tested scale is when it is practically used [21]. Regarding MMT for deltoideus, and MMT for triceps all the tested domains significantly correlated, while for MMT for biceps: Upper extremity, the Sports and physical function, Happiness function and Global function domains showed significant correlations. Our results present satisfactory construct validity when compared with MMT for all tested muscle, where the absence of correlations with the Transfer and basic mobility function as well as the Pain and comfort function domains are not expected in children of this age. Regarding the Transfer and basic mobility function domain’s correlation with MMT, several explanations might be considered. First, this domain includes the motoric action of all body parts, not just arms, and secondly, in this age, children are usually dependent on parents to various degrees. Considering the Pain and comfort function correlation with MMT for this study pathology, the possible explanation might be due to the fact that younger children have less expectations regarding their body’s functioning, including the actions associated with comfort. Furthermore, during the childhood, following the nerve injury, there is reduced tendency for development of peripheral neuropathic pain [22].

For the internal consistency both on the test and retest, all domains except for the Transfer and basic mobility and the Pain and comfort functions of PODCI_SR_ were on an acceptable level. The lower internal consistency for the Pain and comfort function domain was also noticed in the validation of the Dutch [8] and Korean [23] versions of PODCI. When we excluded the question “Did pain or discomfort interfere with your child’s activities?” from the pain and comfort function domain, internal consistency increased to the excellent level. The same observation was noticed in the Korean validation of PODCI [23].

Our study demonstrated excellent test–retest reliability for all PODCI_SR_ domains, thus it can be used in future studies.

There are few limitations to this study. The first refers to the small number of participants, disabling the proper performance of exploratory factor analysis. An additional limitation refers to the age of the studied sample. However, we plan to perform future investigations on a larger number of individuals.

## 5. Conclusions

The Serbian version of PODCI (PODCI_SR_) was successfully translated and transculturally adopted. It has satisfactory temporal stability, construct validity and test–retest reliability as well as relevant internal consistency.

## Figures and Tables

**Table 1:** Serbian version of PODCI raw scores for the test and retest.

Domains	Range Values	Sessions	MV ± SD	95% CI	*p* *
I	0–100	Test	79.63 ± 26.75	71.86–87.39	0.311
		Retest	80.63 ± 25.08	73.34–87.91	
II	0–100	Test	99.25 ± 1.31	98.87–99.63	0.159
		Retest	99.13±1.38	98.72–99.53	
III	0–100	Test	93.15 ± 6.44	91.27–95.02	0.764
		Retest	93.25 ± 6.53	91.35–95.15	
IV	0–100	Test	88.69 ± 13.90	84.65–92.72	0.767
		Retest	88.46 ± 14.35	84.29–92.63	
V	0–100	Test	89.06 ± 22.04	82.66–95.46	0.710
		Retest	88.96 ± 21.61	82.68–95.23	
Global	0–100	Test	90.15 ± 9.60	87.36–92.93	0.752
		Retest	90.25 ± 9.22	87.57–92.93	

MV, Mean Value; SD, Standard Deviation; CI, Confidence Interval; * T test for paired sample.

**Table 2:** Construct validity correlations between Serbian version of PODCI and the Manual muscular test.

PODCI Domains	MMT Deltoideus		MMT Biceps		MMT Triceps	
	ρ	*p* Value	ρ	*p* Value	ρ	*p* Value
I	0.555	<0.001	0.356	0.021	0.420	0.006
II	0.386	0.012	0.279	0.074	0.333	0.031
III	0.450	0.003	0.517	<0.001	0.388	0.011
IV	0.361	0.019	0.151	0.341	0.469	0.002
V	0.650	<0.001	0.565	<0.001	0.580	0.001
VI	0.587	<0.001	0.409	0.007	0.582	<0.001

MMT-Manual Muscular Test; ρ-Spearman’s rho correlation coefficient.

**Table 3:** Internal consistency (Cronbach’s α) for test and retest sessions and test–retest reliability (Intraclass Correlation Coefficient, or ICC) for each PODCI domain.

PODCI Domains	Cronbach’s α		Test–Retest ICC	
	Test	Retest	Values	95% CI
I	0.859	0.846	0.966	0.940–0.981
II	0.168	0.233	0.899	0.826–0.942
III	0.687	0.655	0.932	0.882–0.961
IV	0.107	0.102	0.929	0.877–0.960
V	0.934	0.926	0.996	0.993–0.998
VI	0.838	0.832	0.971	0.949–0.984

ICC, Intraclass Correlation Coefficient; CI, Confidence Interval

**Table 4:** Internal consistency (Cronbach’s α) for test and retest sessions for PODCI domain IV.

PODCI Domain IV	Cronbach’s α	
	Test	Retest
Item 1 excluded	1	1
Item 2 excluded	0.051	0.049
Item 3 excluded	0.051	0.049

**Table 5:** Intercorrelations between Serbian version of PODCI scores.

PODCI Domains	I		II		III		IV		V		VI	
	r *	*p*	r *	*p*	r *	*p*	r *	*p*	r *	*p*	r *	*p*
I	-		0.492	<0.001	0.733	<0.001	0.136	0.356	0.763	<0.001	0.901	<0.001
II	0.506	<0.001	-		0.549	<0.001	0.309	0.033	0.538	<0.001	0.597	<0.001
III	0.727	<0.001	0.443	0.002	-		0.356	0.013	0.767	<0.001	0.846	<0.001
IV	0.142	0.320	0.308	0.033	0.194	0.187	-		0.285	0.050	0.532	<0.001
V	0.852	<0.001	0.462	0.001	0.774	<0.001	0.253	0.083	-		0.804	<0.001
VI	0.899	<0.001	0.596	<0.001	0.780	<0.001	0.542	<0.001	0.851	<0.001	-	

* r, Pearson correlation. The results of the test values are to the right of the diagonal and retest values are to the left of the diagonal.

**Table 6:** Temporal stability due to the patients’ age.

PODCI Domains		MV ± SD
Pair 1	I_TEST_	74.40 ± 26.94
	I_RETEST_	76.12 ± 23.91
Pair 2	II_TEST_	98.92 ± 1.47
	II_RETEST_	98.68 ± 1.52
Pair 3	III_TEST_	92.04 ± 6.37
	III_RETEST_	92.24 ± 6.58
Pair 4	IV_TEST_	90.16 ± 13.26
	IV_RETEST_	89.28 ± 14.04
Pair 5	V_TEST_	89.60 ± 22.31
	V_RETEST_	89.20 ± 21.83
Pair 6	VI_TEST_	88.80 ± 9.83
	VI_RETEST_	88.88 ± 9.06

MV, Mean Value; SD, Standard Deviation.
